# Randomized controlled trial of compressive cryotherapy versus standard cryotherapy after total knee arthroplasty: pain, swelling, range of motion and functional recovery

**DOI:** 10.1186/s12891-024-07310-7

**Published:** 2024-02-28

**Authors:** Aude Quesnot, Simon Mouchel, Salma Ben Salah, Ilana Baranes, Lucas Martinez, Fabien Billuart

**Affiliations:** 1 PT. Laboratoire d’analyse du mouvement, Institut de Formation en Masso- Kinésithérapie Saint Michel / Hôpital de la Porte Verte, 6 Avenue du Maréchal Franchet d’Esperey, Versailles, 78000 France; 2 Département de chirurgie orthopédique, Groupe Hospitalier du Havre, Le Havre cedex, BP24, 76083 France; 3 Unité de Recherche ERPHAN, Versailles, UR 20201, UVSQ France; 4 UFR Simone Veil-Santé, avenue de la source de la Biëvre, Université de Paris-Saclay, Montigny-le-Bretonneux, France

**Keywords:** Total knee arthroplasty, Postoperative rehabilitation, Cryotherapy, range of motion, Swelling, Pain, Function

## Abstract

**Background:**

After total knee arthroplasty (TKA), patients have limited knee range of motion (ROM), trophic changes and pain. Cryotherapy and compression are recommended in the literature, but no study has shown that cryotherapy and compression combined leads to better results than cryotherapy alone. The primary objective was to compare knee ROM after 21 days of rehabilitation post-TKA between patients who underwent rehabilitation with compressive cryotherapy with those who had cryotherapy alone. The secondary objectives were to compare other trophic, pain and functional outcomes.

**Methods:**

Forty patients were randomized into two groups: Standard Cryotherapy (SC = 20, median age 77 years), which applied cold packs along with their rehabilitation; and Compressive Cryotherapy (CC = 20, median age 76 years), which received cold compression. Knee joint’s passive and active ROM (primary outcome) were measured with a goniometer. Knee’s circumference, fluctuation test, pain at rest and during activity, 6-minute walking test (6MWT) and KOOS questionnaire were secondary outcomes. The groups were compared on D1 (baseline) and D21 of rehabilitation. A survival analysis has compared the groups on D1, D8, D15, D21.

**Results:**

All subjects had a significant improvement in all the parameters on D21 relative to D1 (*p* < .05), except for pain at rest (*p* = .065 for CC and *p* = .052 for SC). On D21, the CC group had a significantly larger improvement in the joint effusion (*p* = .002), pain during activity (*p* = .005), 6MWT (*p* = .018) and KOOS (*p* = .004) than the SC group. Based on the survival analysis, the CC group had significantly faster improvement in the joint ROM (*p* = .011 for flexion and *p* = .038 for extension) and knee circumference (*p* = .013) than the SC group.

**Conclusions:**

Both cryotherapy methods improved joint ROM, trophic changes, pain and function. Adding dynamic compression to a cryotherapy protocol provided further benefits: a significantly faster improvement in passive knee flexion ROM, a greater reduction of swelling, and pain during activity. Similarly, walking distance and KOOS questionnaire were significantly better for CC.

**Trials registration:**

The study was registered in the ClinicalTrials.gov database on 14/09/2023 (identifier: NCT06037824).

## Background

Total knee arthroplasty (TKA) is now widely accepted as the preferred treatment for end-stage knee osteoarthritis [1–3]. For several years, the number of TKA procedures done in Europe and the United States (US) has been steadily increasing, with the annual volume of primary TKA procedures reaching 480,980 in 2019 in the US [4]. Shichman et al. [4] contend that this is now the most common orthopedic surgery done in the US. The majority of these TKA procedures are done in women, with a male-to-female ratio of 0.64 in 2019 [5]. Despite the excellent functional outcomes in the short and medium term, better quality of life [1, 2, 6], and a high level of satisfaction, the postoperative period following TKA is key. In fact, during the early postoperative period, patients often have limited joint range of motion (ROM), altered muscle function and edema, with or without joint effusion [1–3]. These can considerably limit function, which can delay rehabilitation and increase the length of hospital stay [1, 3, 6, 7]. Despite the widespread use of enhanced recovery after surgery protocols [3, 8] (multimodal pain management, early rehabilitation, improved anesthesia and surgical techniques), management of the postoperative phase following TKA continues to be a challenge for the care team and patients.

In fact, the international literature recommends incorporating early and routine functional rehabilitation following TKA, with the aims of restoring knee ROM, preventing trophic skin and vascular changes, reducing pain and improving the patient’s independence [1, 3, 9]. Cryotherapy is a nonpharmacological intervention that is recommended and widely used after orthopedic surgery procedure, including TKA [1, 10]. In the TKA context, it consists of external, superficial application of cold fluids to the skin around the knee joint. This technique helps to reduce the temperature inside the joint, slowing nerve conduction velocity and, potentially, the transmission of pain signals [11]. It also reduces peripheral blood flow caused by vasoconstriction, thus decreasing inflammation and local swelling [10, 12]. Ewell et al. [13] suggest that reducing the temperature of the knee joint improves quadriceps activation in patients who have arthrogenic muscle inhibition—often observed after TKA surgery—thereby improving the knee’s joint active ROM.

Several studies have shown the positive effects of external mechanical compression—mainly by bandaging—after TKA surgery, among other physical therapy techniques, on muscular and articular recovery, edema and even pain [14, 15]. External compression is thought to facilitate venous return in the lower limb by improving the effectiveness of the calf muscle pump and moving blood from the superficial venous network to the deep one [16]. Finally, compression is thought to increase interstitial hydrostatic pressure and improve lymphatic microcirculation [16]. Some medical devices providing compression and cryotherapy simultaneously have been around for many years. One of these devices, the Game Ready® system (Coolsystems, Inc. Concord, CA, USA), provides continuous circulation of ice-cold water into different circumferential chambers incorporated into wraps for different joints. The system provides cryotherapy and intermittent dynamic pneumatic compression of 5 to 75 mmHg [17]. It has been shown to be effective at reducing blood loss and analgesic consumption after total hip arthroplasty [18] and TKA [19]. However, no differences were found in terms of the ROM, knee circumference, or various functional tests (6-minute walk test, timed up and go) relative to a group receiving static compression combined with standard cryotherapy. In their recent meta-analysis, Liu et al. [1] found no evidence that any specific cryotherapy technique was better than another following TKA.

The primary objective of this study was to compare knee ROM after 21 days of rehabilitation post-TKA between patients who underwent rehabilitation with compressive cryotherapy with those who had cryotherapy alone. The secondary objectives were to compare other trophic (knee circumferences and joint effusion), pain and functional outcomes (6MWT and Knee Injury and Osteoarthritis Outcome Score). We assume that in the context of postoperative rehabilitation for TKA, there will be a difference between the two groups in favor of the Compressive Cryotherapy (CC) group on the primary and secondary outcomes.

## Methods

### Patients

**Fig. 1:**
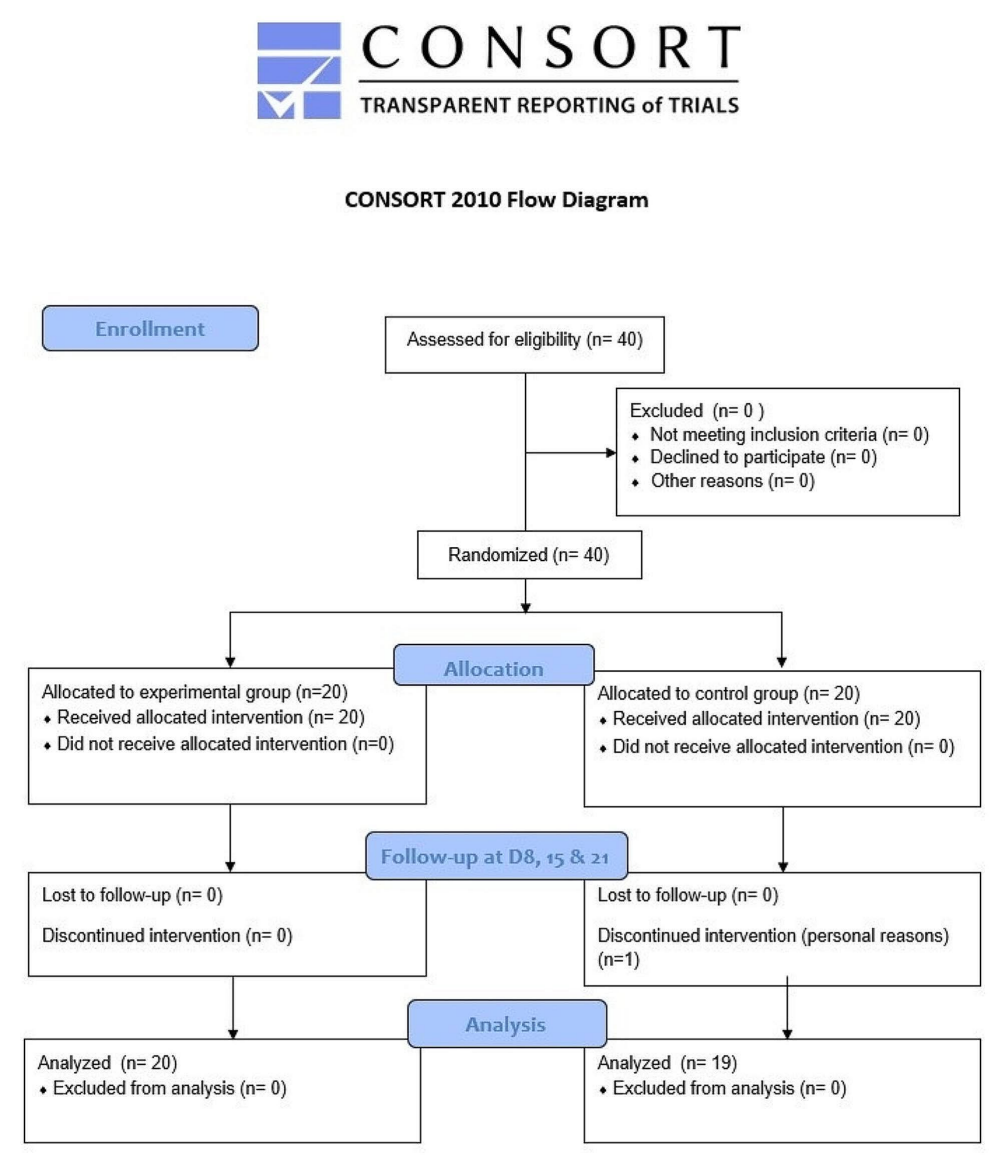
CONSORT 2010 flow diagram for this study

The data were collected between March 11, 2019, and March 25, 2022. Forty patients were randomly assigned after TKA to either the SC group (*n* = 20) or the CC group (*n* = 20) (Table 1; Fig. 1) for their rehabilitation and study treatments. The Ethics Committee of Hôpital de la Porte Verte in Versailles (France) approved the protocol for this study on 01.01.2019. Each subject was given an information letter describing the study; they subsequently provided their informed consent to participate.

**Table 1:** Demographics of the two study groups at baseline (rehab D1)

Data	Compressive cryotherapy		Standard cryotherapy		*P* value
	Median	[IQR]	Median	[IQR]	
Number	20		20		NA
Inclusion (day)	5,57	[3,7 − 6,8]	5.50	[3,6−6,4]	0.233
Women/Men	2/18		7/13		0.108
Age (years)	77.0	[72.0-79.8]	76.0	[71.5–82.5]	0.989
Height (cm)	162.0	[158.0-170.2]	165.0	[157.5–171.0]	0.642
Mass (kg)	80.5	[72.0-84.9]	71.7	[68.3–81.8]	0.160
BMI (kg/m^2^)	31.0	[25.5–33.4]	25.9	[23.7–30.4]	0.116

MED = median; IQR = interquartile range; cm = centimeter; kg = kilogram; NA = Non Applicable; BMI : Body Mass Index; kg/m^2^ = kilograms per meter²; D = Day. Statistical tests: Chi-square test with Yates correction for categorical variables (Women/Men) and Mann-Whitney test for numeric data. Inclusion: in postoperative days.

### Methods

This randomized, controlled, single-blind trial was done at a hospital specializing in physical medicine and rehabilitation.

The inclusion criteria were a primary TKA for knee osteoarthritis diagnosis on radiological and clinical criteria, between 3 and 10 days postoperative, and an age less than 90 years. The exclusion criteria consisted mainly of contraindications to cryotherapy such as cardiovascular disorders, diabetes, circulatory or trophic (wound) problems, Raynaud syndrome and cold urticaria; contraindications to compression such as deep vein thrombosis, ischemic or vascular diseases, carcinomas, and skin lesions. Patients with cognitive disorders were also excluded from this study.

During the study, patients were excluded if they developed an intolerance to cryotherapy or compression, developed surgical complications, withdrew their consent or did not adhere to the rehabilitation protocol (contents and timing of sessions).

The study subjects underwent TKA with a medial parapatellar approach; two different surgeons at two different surgery centers did the operations. All surgeries were done with a tourniquet at the base of the leg. Either Persona® (Zimmer Biomet, Warsaw, IN, USA) or Attune® (DePuy Synthes, Raynham, MA, USA) TKA implants were used. The femoral and tibia implants were cemented.

The same postoperative protocol was used at both surgery centers: paracetamol (1 g/8 hrs. for 1 month), ibuprofen (400 mg/12 hrs.), tramadol for 10 days, gastric mucosal protective agents, low-molecular weight heparin for 35 days, compression socks for 3–4 weeks, weekly blood draws to measured C-reactive protein and platelets. The dressing was changed three times per week by certified nurse with a saline flush. The sutures were removed on Day 18. The patients initially used two crutches or a walker to get around. In accordance with the orthopedic services protocol, all subjects of the study also received cryotherapy with ice packs twice a day for 20 min (morning and evening) from the immediate postoperative period.

Upon discharge from the surgery center, they were admitted to the hospital’s physical medicine and rehabilitation department, where their eligibility for the study was determined. Eligible patients were randomized using a block randomization. This randomization was carried out informatically by a statistician independent of the study. The examiners and physiotherapists were blinded to this randomization.

The day after the randomization (Study Day 1), the subjects started their rehabilitation with a physical therapist who was blinded to the cryotherapy technique used. The study-related measurements were done by an examiner who was also blinded to the cryotherapy technique used. This examiner made the baseline measurements before the treatment was implemented: subject characteristic (Table 1) and the parameters for the study endpoints.

***Knee joint ROM*** was the primary outcome for this study; it was measured with a long-arm goniometer (Saehan Medical Corporation, Chung Buk, South Korea). Passive knee flexion was measured first, then active extension. The patient was placed supine on an examination table. The zero-reference position was the supine patient with knee extended as much as possible. Starting from this position, the patient’s hip was flexed to 90°, then the knee was flexed passively by the examiner. The fixed arm of the goniometer was placed on a line joining the greater trochanter with the lateral femoral condyle; the center was placed on the lateral femoral condyle and the free arm was placed on a line joining the fibular head and lateral malleolus (points marked with skin marker) [20]. The value retained was obtained by subtracting the measurement with the knee flexed from the one with the hip extended on the table. A single measurement was made and retained. The intra-rater reliability (evaluated by the intraclass correlation coefficient) when the patient is supine with the hip flexed is 0.88 [20] with a minimum detectable change (MDC) of 17.6° [20]. Two functional thresholds were set as healing criteria: 90° (ability to sit and walk) and 115° (ability to go down stairs and complete functional recovery) [19]. The patient’s position was the same for active extension. The examiner asked the patient to dorsiflex their ankle, extend their knee as much as possible, then lift their leg while keeping it straight [20]. The examiner recorded the presence of an active extension lag (AEL) using a goniometer according to the same procedure as above (subtracting the measurement of knee actively extended from that of the knee on the table). The intra-rater reliability for active knee extension is between 0.972 and 0.985 [21]. The MDC is 8.2° [20]. Two functional thresholds were also defined as healing criteria: the first was an AEL of 20° or less, which indicates healing progress. The second was 5° or less, which is acceptable for a functional knee [19].

The next set of parameters were the study’s secondary outcomes.

***Knee circumference*** captured swelling of the soft tissues due to an increase in interstitial fluid (edema). The difference in centimeters between the operated and nonoperated knee was measured with a soft measuring tape (Metric, Milan, Italy). The patient was supine on the examination table with the knee fully extended. A measurement was taken at three locations on each leg:

- Base of patella.
- 15 cm above base of patella.
- 20 cm below base of patella.

The intra-rater reliability is 0.99 and the MDC is between 1 and 1.63 cm [22]. A threshold of − 2 cm relative to the baseline measurement on Day 1 was used as a healing criterion.

***Joint effusion***, defined as a postoperative hemarthrosis, was evaluated using the ballottement patellar test [23], with three severity levels: +, ++, +++. With the patient supine, the examiner placed a thumb and finger around the lower portion of the patella and the other hand laced on the upper portion of the suprapatellar bursa. The fingers of the hand nearest the patient’s head exerted light pressure to move any liquid from the prepatellar bursa. The test is positive if the fingers or thumb are pushed apart by the fluid. The more the fingers and thumb are pushed apart, the higher the grade (+). The intra-rater reliability based on the Kappa (k) statistic is 0.37 [24]. In the results section, the test will be expressed as follows: + = 1; ++ = 2; +++ = 3.

***Pain*** was evaluated using the verbal numeric rating scale (VNRS). The examiner asked the patient to quantify his/her pain on a scale of 0 to 10, with 0 being no pain and 10 being the worst pain imaginable. Pain was evaluated at rest (VNRS rest) and during activity (VNRS activity) during the rehabilitation sessions. The intra-rater reliability of the VNRS is 0.95 and the MDC is 1.33 [25]. A threshold of ≤ 3/10 on the VNRS was considered as a healing criterion.

***Walking distance*** was evaluated with the 6-minute walk test (6MWT) [19]. The ICC for this test is 0.94 [25, 26]. In the early postoperative period after TKA, there is no MDC value since the 6MWT has low sensitivity [27]. However, for patients who had a painful and arthritic knee before TKA, the 6MWT has a MDC of 61.3 m [26–28]. Thus, three thresholds were chosen based on the MDC to determine if the difference between two trials of 6MWT was clinically significant. The first threshold was equal to the MDC (61 m). The second was equal to twice the MDC (123 m) and the third was three times the MDC (184 m).

***The patient’s independence*** was evaluated using the 17 items in the “function and ADL” domain of the KOOS out of 68 points. For the KOOS, the intra-rater reliability is 0.90 and the MDC is 15% for the “functional and ADL” domain [29]. The functional threshold chosen as the healing criterion was a value less than 34 (thus less than 50%).

These parameters were measured again on Days 8, 15 and 21 by the same examiner under the same conditions.

During the study, all the subjects received the same rehabilitation protocol consisting of a 45-minute massage and physiotherapy session, five times per week. The rehabilitation protocol involved the following:

- For skin, trophic changes and blood flow: application of compression socks, manual lymphatic drainage and circulatory massages.
- For the knee ROM: multidirectional mobilization of the patella, then assisted-active and then active knee joint flexion and extension, support of the joints above and below the knee.
- For the muscles rehabilitation: elimination of arthrogenic muscle inhibition, relearning how to lock out the knee, progressive strengthening of the muscles around the knee, stretching of the quadriceps and posterior chain muscles (hamstring muscles).
- For functional ability: proprioception work such as single and two-leg balance, weight shifting and resumption of gait pattern, stairs then discontinuation of walking aids.

In addition to rehabilitation, all of the subjects in the study participated in adapted 45-minute physical activity (APA) group sessions, five times per week. The sessions consisted of functional aerobic training, whole body strength training, walking and balance exercises on a walking course.

**Fig. 2:**
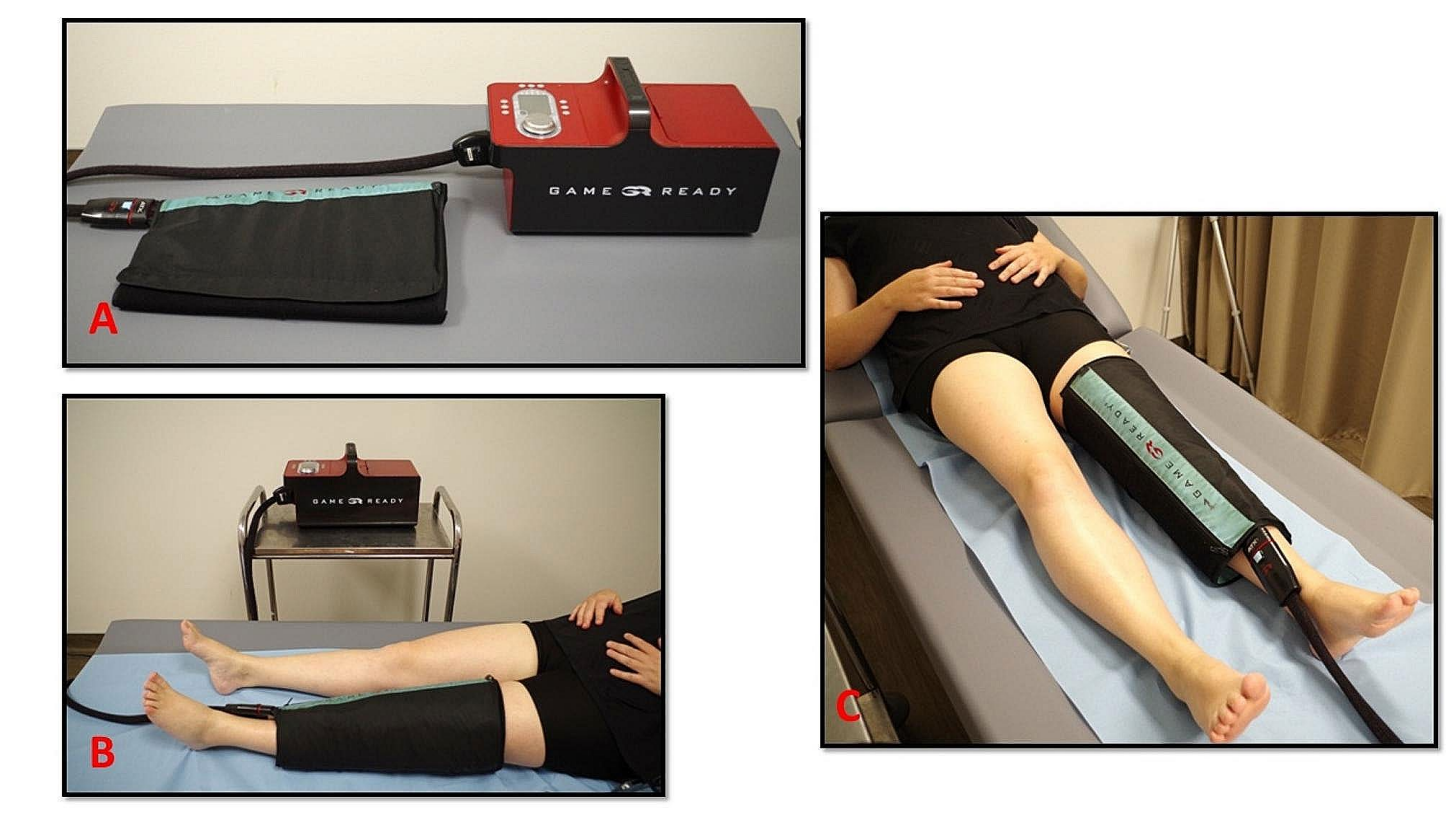
Patient using the game ready® system for compressive cryotherapy. **(A)** device used in the study with the control unit and knee-specific wrap; **(B,C)** patient set-up and use during the study

The two cryotherapy protocols were done outside of the rehabilitation and APA sessions, five days per week throughout the study period. ***Patients in the CC group*** used the *Game Ready®* device (Coolsystems, Inc. Concord, CA, USA). This device consists of a GRPro 2.1® control unit, connector hose and knee wrap developed by the manufacturer (static knee in extension). The patients were installed supine on an examination table with the back rest set at their preferred level. The compression socks were removed, and the knee uncovered. Skin condition was examined. The *Game Ready®* reservoir was filled with 1.5 L water then a block of ice placed in it. After plugging in the connectors, the wrap was placed around the patient’s knee. Once installed, the controller’s parameters were set. The session was 30 min long and the temperature was 4 °C. The pressure was set at *low*, which corresponds to a pressure of between 5 and 15 mmHg. Installation of the device is shown in Fig. 2.

**Fig. 3:**
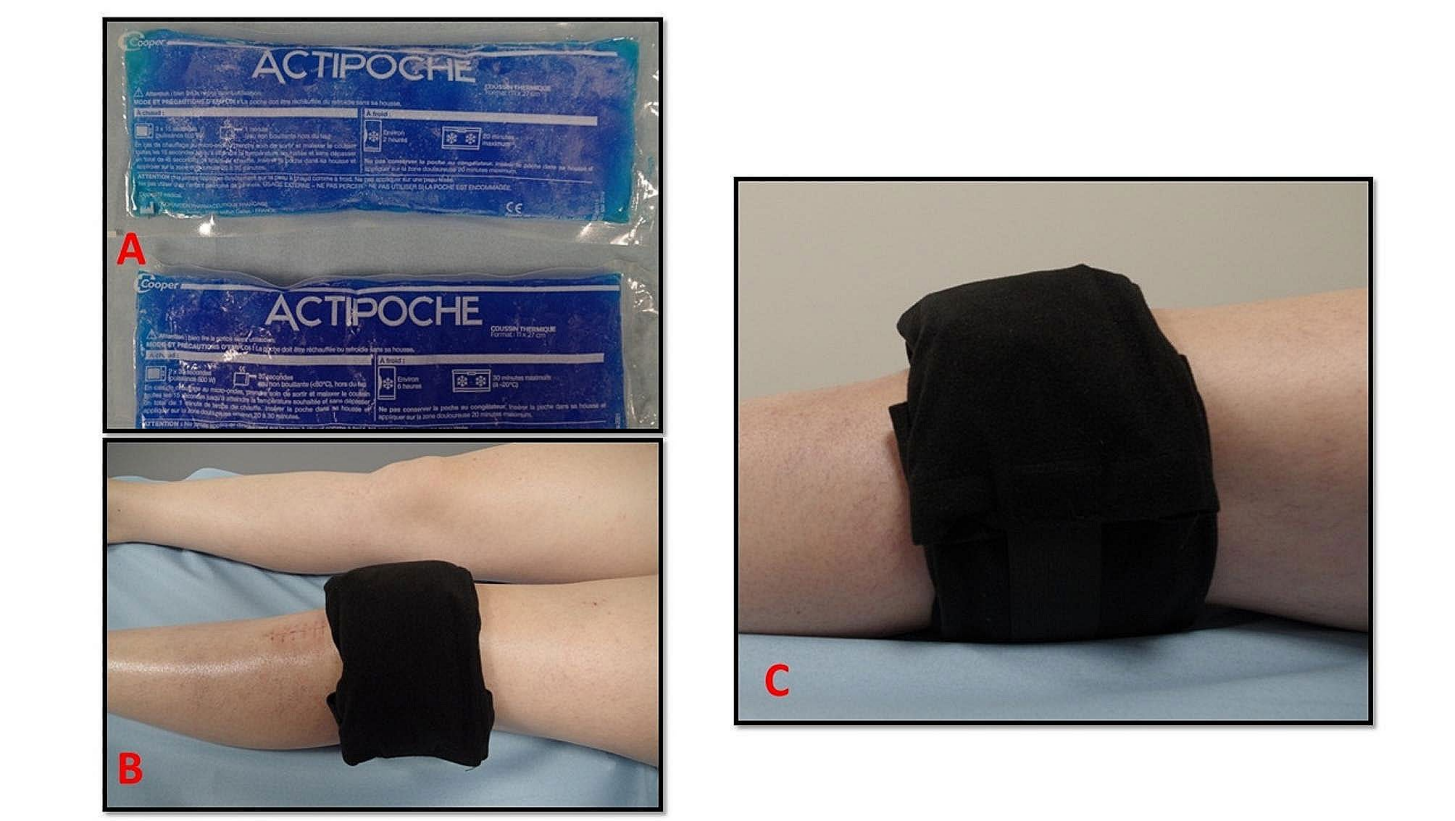
Patient using standard cryotherapy with ice packs. **(A)** the Actipoche® cold packs were placed on the anterior and posterior sides of the knee; **(B, C)** patient set-up and use during the study

***Patients in the SC group*** received standard ice wraps (Actipoche®, Cooper, Melun, France). The compression socks were removed, and the subjects lay supine in their room. Skin condition was examined. The cold packs were placed in non-woven sleeves to protect the skin from burns. The patients applied two ice packs: one on the anterior region of the knee and one on the posterior region of the knee respectively. An elastic bandage (Raucolast®, Lohmann & Rauscher, Rengsdorf, Germany) was applied around the packs to hold them in place. Finally, the device was surrounded by a wet cloth to improve cold conduction. The packs were applied for 30 min, three times per day. The set up is shown in Fig. 3.

### Statistical analysis

Before the study, BiostaTGV was used to calculate the statistical power based on preliminary phase of the study. For a type I error of 0.05 and a statistical power of 0.80, and for observing an increase of 42% in the knee joint’s passive ROM with a pooled standard deviation of 18.92°, 14 patients were needed (7 patients per group). As the study includes many secondary outcomes, the number of subjects was increased to 20 per group.

The statistical results are expressed as median values and interquartile ranges (IQR: Q1–Q3). A level of 0.05 was used to test the statistical hypotheses. The tests were two-tailed with an alpha risk of 0.05 and a beta risk of ≤ 0.02.

Nonparametric statistical tests were used to compare the various groups, since there was no guarantee that the variables were normally distributed (based on the Shapiro-Wilk test). After having tested for normality, The comparisons of the parameters were carried out using a Wilcoxon test.

Homogeneity of the groups was evaluated using the Chi-square test with Yates correction for categorical variables and the Mann-Whitney test for numeric data.

A survival analysis was done using the Kaplan-Meier method. A log-rank test was done with the BiostaTGV software to look for differences in the curves. The remainder of the statistical analysis was done using R software (version 4.2) (Bell Laboratories, Murray Hill, USA).

## Results

The raw data for this study are available as electronic supplementary material. All the study participants followed the same rehabilitation protocol between D1 and D21 in terms of the contents and timing. One subject in the SC group was excluded on D15 after having left the rehabilitation program for personal reasons.

### Homogeneity between study groups

There was no significant difference in the demographics of the two groups (Table 1). At D1 (baseline), the CC and SC groups were not homogeneous in terms of knee circumference at the base of patella (Table 2: *p* = .041) and at − 20 cm (Table 2: *p* = .044).

**Table 2:** Results for the standard cryotherapy group on rehab D1 (baseline) and D21

Parameter	D1		D21		*P* value
	Median	IQR	Median	IQR	
Passive flexion (°)	85.0	[70.0-92.5]	100.0	[92.5-107.5]	< 0.001*
AEL (°)	15.0	[2.5–27.5]	0.0	[0.0-7.5]	0.017*
Circumference at base of patella (cm)	45.0	[43.0-47.5]	42.0**	[41.0-44.7]	0.023*
Circumference + 15 cm from base of patella (cm)	48.5	[45.7–55.7]	44.5**	[42.0-51.2]	0.075
Circumference − 20 cm from base of patella (cm)	36.0	[34.0-38.2]	34.0**	[31.5–37.5]	0.241
Joint effusion (1=+, 2=++, 3=+++)	3.0	[2.0–3.0]	2.0	[1.0–2.0]	< 0.001*
Pain at rest (VNRS)	3.0	[2.0–4.0]	2.0	[0.0-3.5]	0.052
Pain during activity (VNRS)	4.0	[2.5–6.5]	3.0	[1.5–4.5]	0.096
6MWT (m)	194.0	[150.0-265.0]	340.0	[281.7–415.0]	< 0.001*
KOOS (/68)	49.0	[35.0-53.5]	24.0	[15.5–34.5]	< 0.001*

IQR = interquartile range; AEL = Active Extension Lag; VNRS = verbal numeric rating scale; 6MWT = 6-minute walk test; KOOS = Knee injury and Osteoarthritis Outcome Score; * = significant difference; cm = centimeters; m = meters; D = Day

**At D21, the subjects had achieved the threshold value of − 2 cm relative to the initial measurement and defined as healing criterion. Statistical test: Wilcoxon.

### Intragroup comparisons

Intragroup comparisons were made to analyze the changes from D1 to D21 on the various parameters. The treatment effect in the SC group is shown in Table 3. The treatment effect in the CC group is shown in Table 4.

**Table 3:** Results for the compressive cryotherapy group on rehabilitation D1 (baseline) and D21

Parameter	D1		D21		*P* value
	Median	IQR	Median	IQR	
Passive flexion (°)	82.5	[70.0–90.0]	110.0	[95.0-115.0]	< 0.001*
AEL (°)	12.5	[3.7–21.2]	0.0	[0.0–5.0]	0.001*
Circumference at base of patella (cm)	47.7	[45.4–49.9]	42.0	[41.5–43.0]	< 0.001*
Circumference + 15 cm from base of patella (cm)	53.5	[49.5–58.1]	44.7	[44.0–50.0]	0.002*
Circumference − 20 cm from base of patella (cm)	39.2	[36.5–42.5]	36.2	[32.1–39.0]	0.024*
Joint effusion (1=+, 2=++, 3=+++)	3.0	[2.0–3.0]	1.0	[1.0–1.0]	< 0.001*
Pain at rest (VNRS)	3.0	[0.0–5.0]	1.0	[0.0–2.0]	0.065
Pain during activity (VNRS)	4.0	[2.0–6.0]	1.0	[0.0–2.0]	< 0.001*
6MWT (m)	162.5	[150.0-260.0]	439.0	[371.2–480.0]	< 0.001*
KOOS (/68)	40.0	[30.7–60.2]	12.0	[6.7–19.2]	< 0.001*

IQR = interquartile range; AEL = Active Extension Lag; VNRS = verbal numeric rating scale; 6MWT = 6-minute walk test; KOOS = Knee injury and Osteoarthritis Outcome Score; * = significant difference; cm = centimeters; m = meters; D = Day. Statistical test: Wilcoxon.

**Table 4:** *P* values for the SC vs. CC comparisons at rehab D1 (baseline) and D21

Parameter	D1 SC vs. CC	D21 SC vs. CC
	*P* value	*P* value
Passive flexion (°)	0.921	0.186
AEL (°)	0.743	0.308
Circumference at base of patella (cm)	0.041*	0.910
Circumference + 15 cm from base of patella (cm)	0.196	0.612
Circumference − 20 cm from base of patella (cm)	0.044*	0.446
Joint effusion (1=+, 2=++, 3=+++)	0.745	0.002*
Pain at rest (VNRS)	0.549	0.252
Pain during activity (VNRS)	0.756	0.005*
6MWT (m)	0.768	0.018*
KOOS (/68)	0.768	0.004*

In this table, column D1 corresponds to the comparison of SC versus CC for each data measured at D1. Column D21 corresponds to the comparison of SC versus CC for each data measured at D21. Only P values are noted here SC: Standard Cryotherapy; CC: Compressive Cryotherapy; AEL = Active Extension Lag; VNRS = verbal numeric rating scale; 6MWT = 6-minute walk test; KOOS = Knee injury and Osteoarthritis Outcome Score; * = significant difference between SC and CC; cm = centimeters; m = meters; D = Day. Statistical test: Wilcoxon.

In the SC group (Table 3), there was a significant difference (*p* < .05) between D1 and D21 in the following parameters: passive flexion, AEL, knee circumference at base of patella, joint effusion, independence (KOOS) and walking distance (M6WD). There was no significant difference in the circumference at + 15 cm and − 20 cm from base of patella, nor in pain levels.

In the CC group (Table 4), all the parameters except pain at rest (*p* = .065) changed significantly between D1 and D21 (*p* < .005).

### Intergroup comparisons

The two groups were compared on D1 data to determine group homogeneity at baseline. They were compared again on D21 data to test the study hypothesis. The results of these comparisons are shown in Table 2. Only the *p* values are shown in Table 2 since the endpoints at D1 and D21 for each group are shown in Tables 3 and 4. At baseline, there was a significant difference between SC and CC in the knee circumference at the base of patella and at − 20 cm. The knee circumference in the SC group was smaller than in the CC group at the base of patella (Tables 3 and 4: 45.0 cm for SC vs. 47.7 cm for CC) and at − 20 cm (Tables 3 and 4: 45.0 cm for SC vs. 47.7 cm for CC). At day 21, joint effusion was significantly less in the CC group (Table 2: ++ for SC vs. + for CC, *p* = .002), pain during activity was significantly less in the CC group (Table 2: 3 for SC vs. 1 for CC, *p* = .005), and walking distance was significantly greater in the CC group (Table 2: 340 m for SC vs. 439 m for CC, *p* = .018) while the KOOS score was significantly lower in the CC group (Table 2: 24 for SC vs. 12 for CC, *p* = .004).

There were no significant differences between the two groups for passive flexion, AEL, knee circumference at the three locations or pain at rest (Table 2*).*

### Survival analysis

**Fig. 4:**
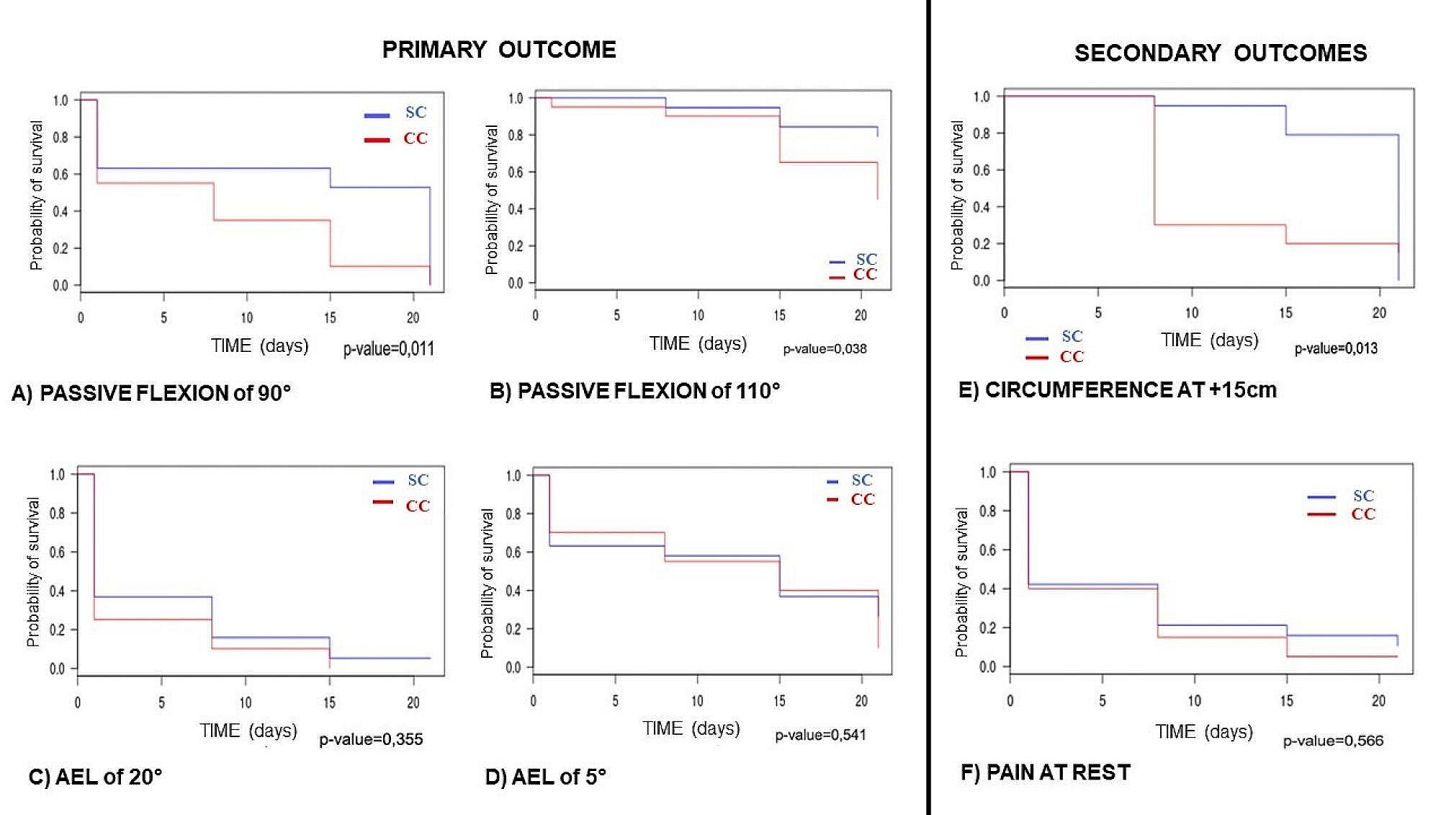
Survival analysis of the study parameters. The Kaplan-Meier curves are shown for **(A)** passive flexion of 90°, **(B)** passive flexion of 110°, **(C)** active extension lag (AEL) of 20°, **(D)** active extension lag (AEL) of 5°, **(E)** knee circumference + 15 cm from the base of the patella and **(F)** pain at rest. SC: Standard Cryotherapy, CC: Compressive Cryotherapy

A survival analysis was done on the parameters that were not significantly different when compared between the two groups (Table 2). The survival curves indicate whether the subjects in one of the two groups exceeded the predefined healing thresholds (see Sect. 2.2). These curves indicated the time (in days) that they took to exceed the threshold, along with the share of subjects who achieved it. A value of 1.0 on the Y-axis means that none of the subjects have achieved the predefined healing threshold. The lower the percentage, the higher the share of subjects who have reached the healing threshold. A value of 0 means that all the subjects have reached the threshold, and thus are considered as having healed. These survival curves also made it possible to statistically compare the rate of progression between groups with *p* < .05 being considered to be a statistically significant difference. The survival curves are shown in Fig. 4.

***For passive flexion***, with a *threshold of 90°* (Fig. 4A), 40% of patients in the SC group had exceeded the threshold at D8 and 50% at D15. In the CC group, 70% had exceeded the threshold at D8 and 90% at D15. This difference between groups was significantly different in favor of the CC group (*p* = .011). With a *threshold of 110°* (Fig. 4B), 20% of the subjects in the SC group reached the threshold on D15 and 25% at D21. In the CC group, 40% reached the threshold on D15 and 60% on D21. There was a significant difference between the two groups in favor of the CC group (*p* = .038).

***For AEL***, with a *threshold of 20°* (Fig. 4C), 85% of subjects in the SC group reached the threshold at D8 and 90% at D15. In the CC group, 90% reached the threshold at D8 and 100% at D15. With a *threshold of 5°* (Fig. 4D), 40% of subjects in the SC group reached the threshold at D8, 65% at D15, and 70% at D21. In the CC group, 45% reached the threshold at D8, 60% at D15 and 90% at D21. There was no significant difference between the two groups (*p* = .541).

***For knee circumference at + 15 cm*** (Fig. 4E), 10% of patients in the SC group reached the threshold at D8 and 20% at D15. In the CC group, 70% reached the threshold at D8 and 80% at D15. There was a significant difference between the two groups in favor of the CC group (*p* = .013).

Lastly, ***for pain at rest*** (Fig. 4F), 80% of the patients in the SC group reached the threshold of 3/10 at D8 and 90% at D15. In the CC group, 90% reached the threshold of 3/10 at D8 and 100% at D15. There was no significant difference between the two groups (*p* = .566).

## Discussion

The current recommendations emphasize the need to start rehabilitation immediately after surgery to address the issues present in the postoperative phase of TKA. Among the existing interventions, cryotherapy is currently recommended despite a lack of consensus on its modalities of use. Thus, the primary objective of this study was to compare knee ROM after 21 days of rehabilitation post-TKA between patients who underwent rehabilitation with compressive cryotherapy with those who had cryotherapy alone. The secondary objectives were to compare other trophic, pain and functional outcomes.

The patients in each group had all the same characteristics, making the comparison between groups valid (Table 1). Also, the patients in each group had identical values on all the parameters measured at D1 (Tables 3, 4 and 2) except for two of the knee circumference measurements (base of patella and 20 cm above), which were higher in the CC group at baseline. This can be explained by the fact that five patients in the CC group had values for these two measurements that were well beyond the third quartile versus only two patients in the SC group, which means that these subjects had more edema immediately postoperative.

Our initial hypothesis that rehabilitation plus CC would improve postoperative joint ROM after TKA more than SC was not confirmed. No matter which cryotherapy method was used, passive flexion improved significantly by D21 (*p* < .001 for SC and CC, Tables 3 and 4). These findings are consistent with previous publications [2, 12, 19, 30–32]. On D21 (Table 2), the CC method was not found to be superior to SC (*p* = .186). However, this is qualified by a survival analysis showing that CC allowed patients to reach the two “healed” thresholds for passive knee flexion significantly faster than SC (Fig. 4A and B): the CC group reached the 90° threshold on D8 and the 110° threshold on D15. This finding may be worth considering as a way to accelerate functional recovery. For AEL, both groups had recovered full knee extension (AEL of 0°) on D21 (*p* = .017 for the SC and *p* = .001 for the CC). On D21, the CC method was not superior to SC (*p* = .308). The survival analysis found no significant difference in how quickly the two groups achieved the so-called “healing” thresholds (Fig. 4C and D). These findings are also consistent with previous publications [19, 31]. Literature reviews and meta-analysis studies [1, 11, 12] on the impact of cryotherapy for improving knee ROM have found a positive effect, but no evidence that one method is better than another. These studies show a link between improved ROM and reduced edema and joint effusion. Our findings on AEL support the hypothesis put forth by Ewell et al. [13] that cryotherapy improves quadriceps activation and thus active extension in patients with arthrogenic muscle inhibition.

In this study, the first to our knowledge to evaluate three different knee circumference measurements, only the CC group improved significantly on all three between D1 and D21, along with joint effusion (Table 4). The SC group had a significant improvement in only two of these four parameters (circumference at base of patella and joint effusion, Table 3). Nevertheless, all the patients in the SC group reached the − 2 cm threshold from the baseline measurement predefined as a “healing” criterion by D21 (Table 3). However, there was no significant difference between the two groups at D21 (Table 2), which is consistent with the studies by Thienpont et al. [31], Sadoghi et al. [32], Su et al. [19] and Thijs et al. [33] on knee circumference at the base of patella. Thus, it seems difficult to show that one cryotherapy technique is better than another based on this parameter, even if the two groups were not identical in the circumference at the base of patella and at − 20 cm on D1 (Table 2). Nevertheless, the survival analysis tends to show that CC improves the circumference at + 15 cm more quickly than SC. In fact, 70% of patients in the CC group had a 2 cm decrease by D8 (Fig. 4E). Relative to our initial hypothesis, CC does not produce greater improvements in trophic and vascular parameters in the early postoperative period after TKA; instead, it improves these parameters more quickly.

There were also several interesting findings in the pain parameters. First, the grades on the pain scales were fairly low from D1 through D21 of this study. Contrary to the studies by Liu et al. [1] and Thienpont et al. [31], we cannot correlated pain levels with analgesic intake as the latter was not recorded during this study. Second, while pain at rest was fairly low from the start, it did not decrease significantly during the study period, no matter which type of cryotherapy was used. These findings are consistent with the results described by Sadoghi et al. [32], Thienpont et al. [31], Kullenberg et al. [34] and Holm et al. [35], which differentiated between various types of pain. This casts doubt on the usefulness of cryotherapy for pain relief. However, the survival analysis (Fig. 4F) found that CC reduced pain at rest to the “healing” threshold faster than SC. As for pain during activity, a significant improvement was seen for the CC group at D21, which was also found by Sadoghi et al. [32] and Kullenberg et al. [34]. This finding was confirmed in the intergroup analysis, which found a significant difference between CC and SC with regard to this parameter (Table 2). The combination of CC and rehabilitation appears to more quickly reduce the pain perceived by patients during activity relative to SC, which partially confirms our initial hypothesis.

Finally, the functional parameters (6MWT and KOOS) were significantly improved in both groups at D21 (Tables 3 and 4). Given that rehabilitation sessions were done several times a week, it is logical that both of these parameters improved. Nevertheless, the CC group had greater improvement than the SC group. We did not find any other studies that analyzed how rehabilitation with cryotherapy affected the KOOS. It appears that rehabilitation plus cryotherapy helps the patient regain independence. Contrary to our study’s findings on walking distance, Su et al. [19] found no significant difference between cryotherapy with static compression and cryotherapy with dynamic compression, yet they did not specify whether both methods were effective at improving this parameter. Given our findings, our initial hypothesis about walking distance appears to be confirmed.

The current study has a number of limitations. First, there was no control group that did not receive cryotherapy. However, it is ethically difficult to withhold cryotherapy during the immediate postoperative period after TKA because its benefits have been proven in previous publications and its use is included in best practices guidelines. Second, the measurements were made by a single examiner who was blinded to avoid interrater variability, especially when measuring ROM as observed by Lenssen et al. [20]. Third, the tourniquet time during the TKA procedure was not factored in. Han et al. [36] contend that tourniquet time affects arthrogenic muscle inhibition, postoperative hemarthrosis, pain and recovery of joint ROM postoperatively. We expect to integrate this variable into future studies to evaluate its impact on the endpoints. The fluctuation test for measuring joint effusion has low reliability, with a Kappa coefficient of 0.37 [37]. Therefore, the results of this test should be interpreted cautiously, even though, to our knowledge, there are no other published rating scales for joint effusion that are more reliable in this specific context. On D1, the two groups differed significantly in knee circumference at the base of patella and at − 15 cm. However, this difference did not prevent us from finding a significant improvement with CC.

## Conclusion

This study is the first to compare postoperative CC to SC after primary TKA. Our findings have several implications. First, they confirm the benefits of cryotherapy, whether compressive or with standard ice packs during the first 21 days of rehabilitation after TKA. With either method, all the studied parameters improved significantly except for pain at rest. Second, CC appears to have some advantages over SC. In fact, CC use improved passive knee flexion significantly faster and decreased joint effusion and pain during activity more and faster than with SC. Similarly, walking distance and recovery of independence during activities of daily living was significantly better when CC was added to the rehabilitation protocol. A multicenter study with a large number of patients could be done to confirm our findings, evaluate how tourniquet time impacts these parameters and the cost-benefit ratio of compressive cryotherapy on the length of hospital stay.

## Data Availability

The datasets used and/or analyzed during the current study are available from the corresponding author on reasonable request.

## Abbreviations

6MWT: 6-Minute Walking Test
AEL: Active Extension Lag
APA: Physical Activity
CC: Compressive Cryotherapy
D: Day
IQR: Interquartile ranges
KOOS: Knee Injury and Osteoarthritis Outcome Score
MDC: Minimum Detectable Change
ROM: Range Of Motion
SC: Standard Cryotherapy
TKA: Total Knee Arthroplasty (TKA)
VNRS: Verbal Numeric Rating Scale
